# The prevalence and break down of narrow anterior chamber angle pathology presenting to a general ophthalmology clinic

**DOI:** 10.1097/MD.0000000000026195

**Published:** 2021-06-18

**Authors:** Nikhil Jain, Rashad Zia

**Affiliations:** a University College Hospitals NHS Foundation Trust; b East Kent Hospitals University NHS Foundations Trust, New Hayesbank NHS Eye Clinic, United Kingdom.

**Keywords:** anterior chamber, glaucoma, narrow angles, prevalence

## Abstract

To approximate the breakdown of narrow anterior chamber angle conditions, on general ophthalmology clinics, in the predominantly white population of the South East Kent region in the United Kingdom.

A review was done of all patients attending a secondary care ophthalmology general clinic over a 3-year period. Patients were assessed with: slitlamp biomicroscopy with indentation gonioscopy; SD optical coherence tomography, Humphrey visual field analyzer, and high frequency ultrasound and categorized into various narrow angle conditions. These were: narrow Van Herrick but open angle; primary narrow angle but nonoccludable; primary angle closure suspect; primary angle closure; chronic narrow angle glaucoma; plateau iris configuration; plateau iris syndrome, and phacomorphic narrow angle.

A total of 14,520 patients were referred to the clinic, of those 10,491 attended and were analyzed. Six hundred seventy four (6.4%) of the patients had some form of narrow angle condition in at least 1 eye. The majority of these patients were at relative low risk of pathology such as nonoccludable narrow angles (359/53.3%) and narrow Van Herrick but open angles (93/13.8%). 8.8% of all the narrow angle patients had primary angle closure suspect or primary angle closure. Plateau iris pathology was seen in 68 (10.1%) of patients with 18 (26%) having confirmed plateau iris syndrome after peripheral iridotomy. Phacomorphic pathology was confirmed in 75 (11.1%) patients.

Narrow angle patients form a significant proportion (6.4%) of those attending general ophthalmology clinic in the predominantly white population in the South East Kent Region of the United Kingdom. The majority of these (67.1%) are at a relatively low risk of developing acute or chronic angle closure glaucoma. Of the remaining patients 8.8% have primary angle closure suspect or primary angle closure and 2.9% have already progressed to chronic narrow angle closure glaucoma. Plateau iris pathology and phacomorphic glaucoma account for the remainder of the presentations.

## 1 Introduction

Glaucoma is the leading cause of irreversible blindness globally and the second leading cause of blindness worldwide after cataracts.^[1]^ The majority of the cases will be due to primary open angle glaucoma with about a quarter being as a result of primary angle closure glaucoma (PACG). In spite of this, it is estimated that they will lead to blindness in a similar number of people worldwide in the year 2020 (5.9 million in open angle glaucoma and 5.3 million in angle closure.^[1]^ With the regards to PACG, one of the most significant predictive factors is a narrow or occludable anterior chamber angle. PACG in particular is more common in those of South-East Asian origin and this is thought to be due to the increased prevalence of narrow or occludable angles in these populations.

Increased prevalence of narrow angles has been shown in populations from Thailand,^[2]^ Burma,^[3]^ China,^[4]^ and other countries from that global region. The range of narrow angle prevalence in these populations various from 10.6% to 36.9%.^[2–5]^ This is significantly higher than the estimates in Caucasian populations which, using the Caucasian-American population as a well-studied example, is thought to be around 3.8%.^[6]^

While the majority of this variation will come from phenotypical differences across populations, some discrepancies will be there due to classification. Historically the anterior chamber angle was graded gonioscopically using either the Scheie^[7]^ or Shaffer^[8]^ system. Van Herrick suggested an alternative method using a slit lamp in 1969^[9]^ and in 1971, Spaeth proposed his grading system which involved elements of the iris insertion, angular approach of the iris, and the peripheral iris contour.^[10]^ In practice, all of these grading systems are used and therefore there can be much heterogeneity as to what defines a narrow angle.

With regards to the UK population, and by extension European populations, the prevalence of PACG has been estimated to be around 0.4% in those over the age of 40 and 0.95% in those over the age of 70.^[11]^ However, there exists little data on the variation of narrow anterior chamber angles presenting to ophthalmology clinics. The term narrow angle can encompass multiple slightly different anatomical variations or pathologies which can convey differing risks to patients. This study was designed to estimate the total number of patients presenting to general ophthalmology clinics with narrow angles and to provide a breakdown of these narrow angles into their various subtypes.

## 2 Methods

A single-phase cross-sectional study was conducted of all patients attending community based secondary care general ophthalmology clinic, in East Kent in the United Kingdom, over a 3-year period from April 1, 2015 to April 1, 2018. All patients were between the ages of 40 to 89. Data was collected from electronic patient records and were not applicable from written patient records. Patients were assessed with slit lamp biomicroscopy, indentation gonioscopy with Spaeth indentation gonioscopic grading, Zeiss© (Germany) SD optical coherence tomography, Humphrey visual field analyzer (Ziess© Forum) and high frequency ultrasound (also referred to as ultrasound biomicroscopy [UBM]). Narrow angle pathology was categorized into the following groups:

1. narrow Van Herrick, but open angle,
2. primary narrow angle but nonoccludable,
3. primary angle closure suspect (PACS),
4. primary angle closure (PAC),
5. chronic narrow angle glaucoma (CNAG),
6. plateau iris configuration (PIC),
7. plateau iris syndrome (PIS),
8. phacomorphic narrow angle.

Patients that made up the narrow Van Herrick, but open angle group were defined as having received a Van Herrick grade 2 or lower on slit lamp biomicroscopy but having no appositional iridotrabecular contact (ITC) on gonioscopy. Nonoccludable primary narrow angles were defined as patients with appositional ITC but less than 180 degrees and intraocular pressure (IOP) less than 21 mm Hg. These 2 groups, given their relative low probability of leading to pathology or visual impairment, were considered low risk.

Those who were stratified as high risk were patients with PAC or chronic narrow angle glaucoma. PACS was defined as appositional ITC greater or equal than 180 degrees with an IOP of less than 21 mm Hg and no glaucoma. Patients’ were defined as having PAC if they had an appositional ITC greater or equal than 180 degrees with an IOP greater than 21mm Hg or with any peripheral anterior synechiae but had no optic nerve head damage. Chronic narrow angle glaucoma (CACG) was defined as characteristic glaucomatous optic nerve head damage with or without raised IOP and an ITC of equal to or greater than 180 degrees. These groups were considered high risk either due to their relative increased probability of developing acute angle closure or potential long-term sight abnormalities.

Three other groups counted were PIS, PIC, and phacomorphic narrow angles. PIC is preoperative conditions where patient has an appositional angle with a flat iris configuration as opposed to the normal “bowing”. Patients with PIC normally have a peripheral iridotomy (PI) to reduce the risk of angle closure, however, should this happen and there still remains evidence of a narrow angle the patient is said to have PIS. For the purposes of this study, a PIC was defined as a plateau iris that reversed after PI and a PIS was defined as a plateau iris persisting after PI and being confirmed on UBM. Finally, phacomorphic narrow angle patients were defined as having an anterior vault height of more than 700 microns on UBM.

Data collection was done in line with hospital ethics procedures and in concordance with the declaration of Helsinki. It was approved by the East Kent Hospitals NHS Trust Ethics committee.

## 3 Results

A total of 14,520 patients were referred to the clinic over the period of time investigated (April 1, 2015–April 1, 2018) 1277 did not attend and 2752 canceled their appointments. This left a total of 10,491 patients that attended the clinic and were fully examined and as such were able to be analyzed in the study.

A total of 674 (6.4%) of the patients were found to have one of the narrow angle conditions listed above. Table 1 summarizes the percentage of number of patients with any of these conditions as a percentage for the total study population.

**Table 1:** Summary of the number and percentage of each narrow angle group out of the total number of narrow angle patients. Also included is the percentage of the total study population made up by narrow angle patients.

Narrow angle pathology	Number of patients	Percentage of total narrow angle cases (%)	Percentage of total study population (%)
Narrow Van Herrick, but open angle	93	13.8	0.89%
Nonoccludable narrow angle	359	53.3	3.42%
Primary angle closure suspect	43	6.4	0.41%
Primary angle closure	16	2.4	0.15%
Chronic angle closure glaucoma	20	2.9	0.19%
Plateau iris configuration	50	7.4	0.48%
Plateau iris syndrome	18	2.7	0.17%
Phacomorphic narrow angle	75	11.1	0.71%
Total	674	100	6.42%

Subsequent subgroup analysis was performed on the 674 narrow angle patients to ascertain the variation in pathology. The largest group was the nonoccludable narrow angle group which had 359 (53.3%) patients. The next largest group was the narrow Van Herrick but open angle group with 93 (13.8%); this meant that a total of 452 (67.1%) patients presented with relatively low risk of acute angle closure or angle closure glaucoma.

**Figure 1:**
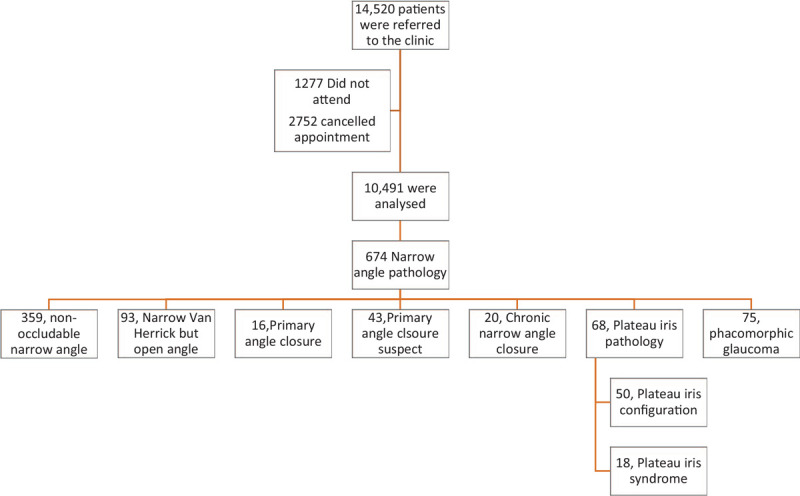
Summary of the breakdown of narrow angle pathologies as a percentage of the whole population and of the total number of narrow angles.

Fifty (7.4%) patients had PIC and 18 (2.7%) had PIS. Phacomorphic narrow angle was confirmed in 75 (11.1%) of patients and of the remaining patients: 16 (2.4%) had PAC, 43 (6.4%) had PACS and 20 (2.9%) had CACG. This is summarized in Figure 1.

## 4 Discussion

Using publicly available census data the percentage of the UK population that is classified as “white” is 86%, this is lower than the 94% in the East Kent population.^[12]^ These are both derived mainly from “white British” people while the remainder are “white other” usually referring to people of European descent also. The overwhelming majority of patients that were referred to the clinic were therefore Caucasian and of the 674 patients that had some variety of narrow angle all were white. This means that this data can only be used to estimate the breakdown of narrow angles in Caucasian populations and for no other group as the samples were not larger enough.

As described above the prevalence of narrow angles in the Caucasian-American population is around 3.8%, which may not reflect our value of 6.4% as typical workload in a general clinic. The sources of this discrepancy could be due to numerous reasons. Firstly, there could be subtle environmental or behavioral discrepancies between Caucasian-Americans and the White UK population that result in the difference. Secondly, the sample size used in this study was much larger than in other such studies and hence the difference could arise from a larger more representative sample being analyzed. Thirdly, as mentioned above, there are many alternative ways to measure and record whether an angle is narrow or not and as such discrepancies can come from using different grading systems. Finally, the data in this study was all collected on patients that had already been referred for 1 reason or another to general ophthalmology clinic. This means that it can reliably be said that narrow angles form 6.4% of the workload of ophthalmology clinics however this is likely a higher number than the general population as those people without at least some suspicion of ocular pathology have already been excluded by way of not warranting referral.

While this study suggests a higher prevalence of narrow anterior chamber angles in white populations than previous studies this is till comfortably below the range in the above mentioned South East Asian populations (range 10%–65%).^[2–5]^ It has also been well established that the majority of glaucoma (about 90%) is due to primary open angle glaucoma in Caucasian populations while this is reversed and about 90% of glaucoma in certain Asian populations is secondary to PACG.^[13]^ The value of 6.4% of the white UK population corresponds to a prevalence of 0.4% of PACG in this population, whereas the estimates of narrow angles in the Chinese population is around 36%^[4]^ which corresponds to a disproportionately low prevalence of PACG of 1.10%.^[14]^ This would be in accordance with previously established theories that narrow or occludable angles are a predictor for PACG^[4]^ but not the single best predicator.

With the question of workload established the more difficult question is how to best manage these patients. There is scant literature on the exact level of risk of developing pathology secondary to having an occludable angle alone. A study of European derived populations followed up 129 patients with narrow angles and pachymetry measured anterior chamber depth of less than 2 mm. The data showed that 8 patients (6.2%) developed acute angle closure glaucoma and 17 (13.2%) developed angle closure but with no symptoms, rise in IOP, or optic disc disease.^[15]^ It has been suggested therefore that these patients may benefit from a prophylactic peripheral iridotomy (PI) to reduce risk.^[16]^ Indeed, the Zhongshan angle prevention trial, a well-designed randomized controlled trial on the Chinese population, concluded that the risk reduction by offering prophylactic YAG PI to angle closure patients is 0.38% per year and the number needed to treat is 263 per year.^[17]^ Hence a large number of patients need to be treated to reduce the incidence of acute angle closure.

In our study, we identified that the majority of the narrow angle patients were of low risk (narrow angle but unoccludable or narrow Van Herrick but gonioscopically open angle) and it is probable that these patients would not require a PI without any other evidence. Common practice however is to offer all of these patients a peripheral iridotomy. This is not relevant for all members of the high-risk group though, as plateau iris patients have already received a PI and still maintain a high risk of acute angle closure glaucoma and need appropriate follow up.^[18]^ Phacomorphic narrow angle patients would likely benefit more from prompt phacoemulsification and intraocular lens (IOL) insertion than a prophylactic PI. Further to this the results of the EAGLE trial, a multicenter randomized control trial to estimate the effectiveness of clear lens extraction vs peripheral iridotomy, suggested that phacoemulsification with subsequent IOL insertion be the preferred first line management in those with primary angle closure and PACG.^[19]^

## 5 Conclusions

Narrow angle patients form a significant proportion (6.4%) of those attending general ophthalmology clinic in the predominantly white population in the South East Kent Region of the United Kingdom. The majority of these (67.1%) are at a relatively low risk of developing acute or CACG. Of the remaining patients 8.8% have primary angle closure suspect or primary angle closure and 2.9% have already progressed to chronic narrow angle closure glaucoma. Plateau iris pathology and phacomorphic glaucoma account for the rest of the presentations.

## Author contributions

**Supervision:** Rashad Zia.

**Writing – original draft:** Nikhil Jain.

**Writing – review & editing:** Nikhil Jain.
